# Nine quick tips for open meta-analyses

**DOI:** 10.1371/journal.pcbi.1012252

**Published:** 2024-07-25

**Authors:** David Moreau, Kristina Wiebels

**Affiliations:** School of Psychology and Centre for Brain Research, University of Auckland, Auckland, New Zealand; SIB Swiss Institute of Bioinformatics, SWITZERLAND

## Abstract

Open science principles are revolutionizing the transparency, reproducibility, and accessibility of research. Meta-analysis has become a key technique for synthesizing data across studies in a principled way; however, its impact is contingent on adherence to open science practices. Here, we outline 9 quick tips for open meta-analyses, aimed at guiding researchers to maximize the reach and utility of their findings. We advocate for outlining preregistering clear protocols, opting for open tools and software, and the use of version control systems to ensure transparency and facilitate collaboration. We further emphasize the importance of reproducibility, for example, by sharing search syntax and analysis scripts, and discuss the benefits of planning for dynamic updating to enable living meta-analyses. We also recommend publication in open-access formats, as well as open data, open code, and open access publication. We close by encouraging active promotion of research findings to bridge the gap between complex syntheses and public discourse, and provide a detailed submission checklist to equip researchers, reviewers and journal editors with a structured approach to conducting and reporting open meta-analyses.

Systematic reviews are comprehensive syntheses of evidence that aim to answer a specific research question by systematically identifying, appraising, and summarizing all relevant studies on a topic. Unlike traditional narrative reviews, systematic reviews follow a rigorous and transparent methodology to minimize bias and ensure the reproducibility of their findings.

Meta-analysis—a statistical technique often used within systematic reviews to quantitatively combine and analyze the results from multiple individual studies—has emerged as a cornerstone methodology in scientific research, enabling scholars to synthesize results from multiple studies to draw comprehensive conclusions, with greater statistical power and generalizability than individual studies alone [1]. Pooling data across sources, meta-analyses can uncover trends and insights that might not be apparent in single studies, thereby providing a more reliable foundation for cumulative science, theory building, and evidence-based policy [2].

While systematic reviews and meta-analyses are closely related, not all systematic reviews necessarily include a meta-analysis: some reviews may focus on synthesizing qualitative or descriptive data, while others may not find sufficient homogeneity among the included studies to justify conducting a quantitative meta-analysis. However, when appropriate, meta-analyses can add significant value to systematic reviews by providing a quantitative synthesis of the available evidence.

In parallel with the steady rise of meta-analysis, the open science movement has sought to improve the transparency, reproducibility, and accessibility of scientific research. Open science principles advocate for the sharing of data, materials, and methodologies so that findings can be verified and built upon more easily by other researchers [3]. Studies have shown that the adoption of open science practices can enhance the credibility of scientific findings and foster greater innovation and collaboration within the research community [4]. Despite the clear synergy between the goals of meta-analysis and open science, integrating these practices remains a challenge. As such, clear guidelines might be helpful to navigate the complexities involved [5].

Here, we bridge the gap between meta-analysis methodology and open science principles by proposing 9 quick tips for open, transparent meta-analyses. These tips, summarized in Fig 1, are intended to help researchers design, conduct, and publish meta-analyses that adhere to the highest standards of openness and transparency, ensuring that their findings can be trusted, replicated, and built upon by the scientific community. We also provide a checklist to help researchers, reviewers, and journal editors implement these guidelines in practice (https://osf.io/k8aqx/).

**Fig 1:**
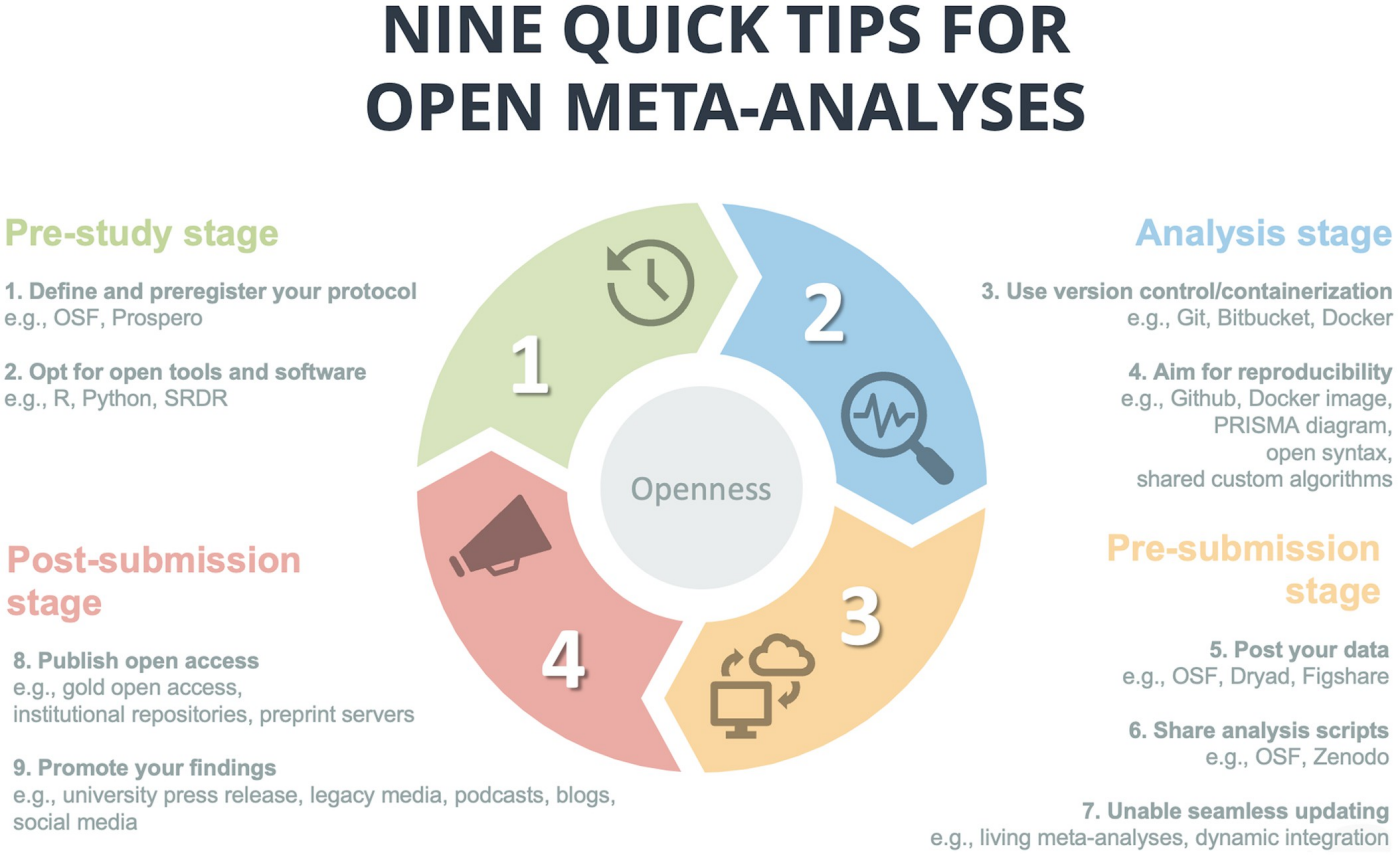
Nine quick tips for open meta-analyses.

## Tip #1: Define and preregister your protocol

Meta-analysis serves as a powerful tool to synthesize data from multiple studies, but its validity and robustness is contingent on establishing a well-defined protocol at the onset of a project [6]. Defining and preregistering a clear protocol before conducting a meta-analysis helps safeguard against potential biases and ensure the transparency and reproducibility of the research process. This includes outlining the scope of the meta-analysis, its rationale, main and secondary hypotheses, and primary and secondary outcomes, specifying the procedure for literature search, study selection, data extraction, determining the study inclusion and exclusion criteria, and deciding on assessments for the quality and risk of bias in included studies. Typically, protocols should also outline the analysis plan, including statistical models, approaches for handling data heterogeneity, publication bias, and sensitivity analyses [7–9].

Once a protocol has been defined, preregistering it ensures transparency, accountability, and helps with its reproducibility [8,9]. Preregistration entails the a priori documentation of the research plan before the analysis begins, solidifying the methodological framework and analytic strategies [10]. This process requires detailing the study’s objectives, hypotheses, methodology, and statistical analysis plan in a time-stamped registry that is publicly accessible [11]. Common platforms for preregistering meta-analyses include PROSPERO (https://www.crd.york.ac.uk/prospero/) or the Open Science Framework (OSF; https://osf.io/). It is important to note that PROSPERO is a closed protocol registry that primarily accepts certain types of systematic reviews, such as those related to health and well-being, and prioritizes the registration of reviews conducted by UK-based researchers. In contrast, OSF is an open platform that allows researchers to register their projects, as well as share research data, materials, and analysis tools. While both platforms serve the purpose of preregistration, OSF offers a more flexible and open approach for researchers across various disciplines, for example, via predefined templates such as the Generalized Systematic Review Registration. Irrespective of the specific platform one chooses, preregistration serves as a declaration of the analytical roadmap, which enhances the study’s credibility and reproducibility [8]. It also helps differentiate between confirmatory and exploratory analyses, which is important for interpreting the findings accurately and for readers to assess the extent to which the results were hypothesized in advance [12].

Although preregistration may seem redundant in the context of meta-analysis, given that data is retrospectively collected from existing studies, it nevertheless serves several critical functions. Preregistration acts as a public commitment to a specific analysis plan, which enhances the credibility of the research by preventing undisclosed, ad hoc changes to the methodology that could be influenced by the data outcomes [12]. This in turn provides a safeguard against the introduction of bias, especially the kind that may arise from selective reporting or outcome switching after interim results are known. While it does not prevent post hoc decision-making per se, preregistration thus makes it easier for readers to detect deviations from the preregistered plan, which can help identify potential sources of bias introduced by such deviations [10–12]. Importantly, deviations from the preregistered protocol should be reported and justified in the final publication to maintain the integrity of the research process [9].

The commitment to preregistration also aligns with the FAIR principles, ensuring that the research plans and protocols are findable, accessible, interoperable, and reusable, thereby contributing to the collective effort of fostering open science [13]. It is a proactive measure that communicates to the scientific community the integrity of the research process and the authenticity of the research intent [14]; as such, preregistration embodies a cornerstone practice in open meta-analysis, setting the stage for studies that are not only methodologically sound but also publicly accountable.

## Tip #2: Opt for open tools and software

The credibility and trustworthiness of meta-analyses is greatly enhanced when researchers opt for open tools and software, which facilitate transparent, replicable, and verifiable research practices [15]. Open tools and software are not only free to use, they also allow others to examine and validate the underlying code, ensuring that the methodological processes are laid bare for scrutiny [16]. Moreover, open tools and software foster code reuse, allowing researchers to build upon existing work rather than starting from scratch, accelerating progress and avoiding duplication of effort.

Openness can be promoted at every step of a meta-analysis, from data extraction to the final statistical analysis. Using open-source statistical software like R [17], with packages like *meta* [18], *metafor* [19], and *revtools* [20] for meta-analysis, or Python [21], with generic packages for meta-analysis like *PythonMeta* [22] and *PyMARE* [23], or specialized packages such as *NiMARE* [24] for neuroimaging meta-analyses or *AutoGDC* [25] for DNA methylation and transcription meta-analyses, enables researchers to share their code, thereby providing a transparent audit trail from raw data to results. Other open-source options include JASP [26] and jamovi [27], both of which are full statistical software that include meta-analysis modules.

The recommendation also extends beyond statistical analysis, to embrace free tools for systematic review management. The Systematic Review Data Repository (www.srdrplus.ahrq.gov) [28], developed by the Agency for Healthcare Research and Quality (AHRQ), is an online repository and data management platform specifically designed for conducting systematic reviews. It provides structured forms for extracting data, assessing risk of bias, and tracking the review process, while enabling secure collaboration among review teams. Similarly, Rayyan (www.rayyan.ai) [29] is a web-based application that streamlines the screening of literature search results for systematic reviews and meta-analyses. It facilitates collaborative screening, allowing multiple researchers to independently evaluate studies in a blinded manner, while tracking screening decisions and conflicts. These platforms can help researchers transparently manage their review process and share their progress with the community. Paid subscription-based alternatives exist (e.g., Covidence; Rayyan also includes paid plans), with additional functionalities such as tighter integration with reference management software or more advanced project management capabilities; however, in most cases open tools are perfectly adequate. Adherence to open tools is not a mere technicality, but a principled stand for open science, which often symbolizes a researcher’s commitment to collaborative progress and to the democratization of knowledge [30].

Moreover, open-source can extend to version control systems, which allow for meticulous tracking of changes and collaborative input on the analytic scripts [31] and to software containers, which further enhance the reproducibility of meta-analyses. We turn to these tools with our next tip.

## Tip #3: Use version control or containerization

In the context of open meta-analyses, version control systems help maintaining transparency, accountability, and collaborative integrity. Beyond its wide use in software development, version control is an indispensable asset for researchers managing the complexities of meta-analytical workflows [32]. Services such as Git, an open-source version control system (www.github.com; see also Bitbucket for an alternative: www.bitbucket.org), when integrated with online platforms like GitHub, provide a transparent mechanism to document the evolution of a project, offering snapshots of every stage in the project’s lifecycle [33]. This allows for the identification of who made particular changes, when these alterations were implemented, and why certain methodological adjustments were necessary, which is crucial in multi-contributor projects where coordination and clarity are paramount [34,35]. In addition, platforms like OSF offer the capability to update preregistrations, allowing researchers to document and justify any deviations from the initially preregistered protocol. This feature complements version control systems by providing a centralized location to track and explain changes to the preregistered plan, further enhancing transparency and accountability throughout the research process.

Incorporating the use of containers, such as Docker (www.docker.com) or Singularity (www.sylabs.io), can further enhance the reproducibility and portability of meta-analyses. Containers encapsulate the analysis environment, ensuring that all necessary computational tools, libraries, and dependencies are bundled together [36]. This guarantees that the analysis can be reliably replicated across different computing environments and across software releases, reducing the “it works on my machine” phenomenon that can hinder reproducibility [36]. The adoption of containers aligns seamlessly with version control practices. For example, a Dockerfile—essentially a blueprint for building a container—can be version-controlled alongside analysis scripts [37]. This allows for the entire computational environment to be versioned, shared, and archived, providing a more robust mechanism for replicating and verifying research findings [38].

With the implementation of version control and container technology, meta-analyses become more accessible and transparent. Researchers can not only track the iterative progress of their work but also ensure that their computational analyses are reproducible by anyone, anywhere. This extends to managing contributions across various collaborators, enabling the synthesis of insights while preserving the individual contributions of each team member [39], ensuring that intellectual input is accurately credited, and fostering a culture of recognition and respect within research teams. Furthermore, such approach supports reproducibility and data provenance, allowing future researchers to revisit and build upon past work with confidence in its veracity [40]. As such, it acts as a safeguard against the loss of data and analysis versions, proving indispensable in times of unexpected disruptions or when reverting to previous iterations is necessary [41].

## Tip #4: Aim for reproducibility

A natural outcome of version-control systems, especially implemented as containers, is reproducibility [36]—a hallmark of credible scientific research, particularly critical in the context of meta-analyses. Aiming for reproducibility mandates meticulous documentation of all aspects of the research process to ensure that other investigators can replicate the findings and trust their validity [42]. This extends to providing explicit search strategies, including search syntax and dated search results from databases, which are fundamental for enabling others to reproduce the literature search with precision [6].

The detailed recording of search strategies should include the databases searched, the full electronic search strategy for at least 1 database, the date last searched, and any limits applied, as advocated by the PRISMA guidelines [6]. Ideally, researchers should include the exact search syntax used, tailored for each database, to account for variations in indexing terms and functionalities across different databases [43]. The selection process of studies, including screening, eligibility criteria, and the reasons for excluding particular studies, is typically summarized in a PRISMA flow diagram; this enables others to understand decision-making and evaluate the potential for selection bias [44]. Sharing dated search results from databases enhances transparency, as it accounts for the dynamic nature of databases where the availability of studies may change over time [45].

Furthermore, researchers should extend reproducibility efforts to data extraction and analysis phases by sharing their extraction forms, code, and any custom algorithms used [30], in a process that reinforces the credibility and utility of the findings [46,47]. In this context, transparent reporting involves a detailed account of the search strategy, including search terms, databases, date ranges, and any restrictions used. Researchers should also provide the search syntax for each database searched to enable replication [48]. Reporting should typically include the screening process, selection criteria, and the flow of information through the different phases of a meta-analysis, often depicted with a PRISMA flow diagram [7].

Risk of bias assessment is a fundamental step in meta-analyses to evaluate the methodological quality of included studies and detect potential sources of bias that may affect the validity of findings. To promote transparency and reproducibility in this process, researchers should prioritize open tools and instruments for assessing risk of bias. The Cochrane Risk of Bias tools (RoB 2 for randomized trials [49] and ROBINS-I for non-randomized studies [50]), freely available online (https://www.riskofbias.info), provide structured frameworks and clear guidance for bias appraisal. These tools can help streamline the risk of bias assessment, ensure methodological rigor, and enhance the replicability of their quality evaluations.

Ideally, researchers should also document all decisions made throughout the study, including the rationale behind the exclusion of certain studies and the methods used for data extraction and risk of bias assessment. This extends to the statistical methods and any sensitivity analyses performed, with justifications for the models and parameters [51], and to any deviations from the preregistered protocol. While journal articles may have limited space for such technical details, researchers should take advantage of supplementary materials or appendices to comprehensively document their decision-making processes, analytical choices, and any deviations from the preregistered plan. These supplementary files can be hosted alongside the main article or in open repositories, ensuring that the complete methodological details are openly accessible and citable. Together, these steps toward reproducibility help the reliability of meta-analyses, but also contribute to the collective trust in the findings presented within the scientific community.

## Tip #5: Post your data

Posting data is an imperative principle in the domain of open meta-analysis, fostering a collaborative scientific environment where data are not only shared but also are made accessible for scrutiny and reanalysis [52]. Open data involves making the raw data collected from studies, as well as the extracted data used for meta-analytic computations, available in a public repository [53]. In the context of meta-analyses, this typically includes: effect size estimates (e.g., standardized mean differences, correlation coefficients, odds ratios) and associated statistics (sample sizes, standard errors) extracted from each included study; study-level characteristics or coding for potential moderator variables; and risk of bias assessments or ratings of study quality. Choosing the right repository is crucial; it should guarantee the longevity and accessibility of the data. Repositories like OSF, Dryad (www.daradryad.org), or Figshare (www.figshare.com) provide DOI-linked storage, ensuring that the data can be properly cited and linked back to the original research [54].

When choosing data-sharing platforms, researchers should consider long-term sustainability and durability. While popular platforms like GitHub offer convenient collaboration and versioning features, it is important to recognize that they are commercial entities subject to potential changes in business interests or ownership. For long-term preservation and access, researchers may want to prioritize platforms with explicit commitments to data archiving and long-term access plans. For example, OSF has contingency plans in place to ensure that data and materials hosted on their platform are preserved for a minimum of 50 years. Alternatively, researchers could adopt a hybrid approach, using platforms like GitHub for active version control and collaboration during the research process, but also archiving snapshots of their repositories and scripts in dedicated, long-term preservation.

The benefits of posting data are multifaceted: it increases the trust in the findings, enables other researchers to conduct secondary analyses or meta-analyses, and contributes to the reduction of research waste by avoiding the duplication of efforts [55]. When posting data, researchers must ensure that it conforms to all applicable privacy regulations and ethical standards [56]. Ideally, the data should be accompanied by detailed metadata, data dictionaries, and any relevant scripts or algorithms used to process the data. This ensures that other researchers can understand and replicate the analysis [57], in a commitment to transparent and reproducible science that upholds the integrity of the research and advances the collective knowledge within the field.

In addition to posting the meta-analytic data, researchers can also leverage open data repositories to access and extract data from the primary studies included in their meta-analysis whenever possible. Many journals and funders now require authors to make their raw data publicly available, offering opportunities for meta-analysts to obtain original datasets directly. Repositories such as OpenNeuro (https://openneuro.org) for neuroimaging data or GenBank (https://www.ncbi.nlm.nih.gov/genbank/) for nucleotide sequences can be invaluable resources for accessing primary data directly, which can reduce inaccuracies from manual extraction, enable more comprehensive data synthesis, and facilitate novel exploratory analyses.

## Tip #6: Share analysis scripts

Open meta-analyses hinge on the replication and validation of research findings. Sharing analysis is an essential aspect of reproducibility, enabling others to verify results and conduct further analysis [58,59]. When researchers share their analysis scripts, they facilitate a deeper understanding of the methods used in the research, which can help identify potential issues and improve upon the proposed methods [60]. This practice should be standard, with scripts shared via repositories such as GitHub or Zenodo (www.zenodo.org), which provide DOIs for each release to ensure that the exact scripts used can be cited [61].

To follow good practices, scripts should be well commented, detailing the purpose and function of each section of code. This is critical as it provides context to the scripts, making them understandable to others who may not be familiar with the specific project or the coding language used [62]. Furthermore, sharing scripts encourages efficiency and collaboration as it allows others to build on existing work rather than starting from scratch [41]. Researchers are encouraged to license their scripts in a way that permits reuse and modification, such as through permissive licenses like the MIT or GNU General Public license, or using a Creative Commons Attribution 4.0 International (CC BY 4.0) license. The latter permits the reuse and modification of the work, while explicitly requiring attribution to the original authors. This can ensure that researchers’ intellectual contributions are properly acknowledged while still promoting the open sharing and collaborative development of their work.

## Tip #7: Enable seamless updating

Traditional meta-analyses can rapidly become outdated as new research accumulates. While methodically rigorous, static reviews are snapshots that reflect the evidence available up to the point of their completion, and the lack of subsequent integration of new data can lead to periods where the meta-analysis is not reflective of the current state of evidence [47].

In response to this limitation, living meta-analyses are a form of systematic review that are regularly updated as new evidence becomes available. This approach ensures that the meta-analysis remains current and continuously reflects the latest data on a topic [63]. The structure of such a living document requires a rigorous initial protocol that specifies not only the methodology for the initial review but also the strategy for ongoing evidence surveillance, criteria for determining the significance of new data, and the process for their assimilation into the existing meta-analytic framework. Enabling seamless updating, particularly in the form of living meta-analyses, is especially valuable in areas where research evidence is rapidly evolving, as it can more accurately inform timely decision-making in clinical practice, policy, and further research.

The successful implementation of living meta-analyses is contingent on meticulous planning for data management and analysis update. This includes predefined methods for literature search updates, explicit inclusion and exclusion criteria, and robust statistical strategies capable of integrating new data without compromising the validity of the meta-analysis [64]. It also entails setting thresholds for what constitutes significant new evidence that warrants an update, thereby maintaining the balance between the currency of the analysis and the practicality of the update process.

Importantly, while committing to the implementation of living meta-analyses may not be feasible for all research teams, it is still beneficial to organize data and code in a way that enables future updates and maintains the potential for the review to evolve into a living document. One key consideration is the structured organization and documentation of data extraction processes and analytical pipelines. Researchers should strive to create modular and well-documented code that can be easily adapted to incorporate new data as it becomes available. Version control systems can help track changes and facilitate collaborative updates, ensuring that the review remains a living, evolving entity, while the use of containerization technologies can help encapsulate the entire computational environment, for seamless updating (see also Tip #3). Another important aspect is the use of robust data management practices that allow for the efficient retrieval and integration of new study information. This may involve the use of relational databases or other structured data storage solutions, as well as the development of standardized data dictionaries and metadata schemas.

## Tip #8: Publish open access

Open access publication ensures that the results of research are accessible to all, without paywall restrictions, enabling broader dissemination, greater visibility, and increased citation and use of the work [65]. Open access can take various forms, including diamond/platinum open access, where articles are free to both authors and readers; gold open access, where the final published article is immediately open for all to read and use; bronze open access, where the article is freely accessible but without an explicit license; and green open access, which involves self-archiving a version of the article in a repository [66]. Given their relevance to guide practice and policy, the imperative for open access is even stronger in the case of meta-analyses, as it underpins the drive for informed decision-making in various sectors [67].

Researchers are encouraged to consider open access options when selecting a journal for submission, bearing in mind that many funding agencies now mandate open access publication as a condition of their grants [68]. However, caution should be exercised when considering open access options, as the landscape includes predatory publishers who exploit the open access model for profit while lacking robust peer review and editorial processes; resources like Cabell’s Predatory Reports (https://www2.cabells.com/about-predatory) or the Directory of Open Access Journals (https://doaj.org/) can help researchers identify reputable open access journals and publishers.

It is also important to acknowledge that the costs associated with gold open access publication can pose significant challenges for researchers, particularly those in the Global South or from institutions with limited funding resources. The article processing charges (APCs) levied by many open access journals can be prohibitively expensive, creating inequities in the ability to publish and disseminate research findings openly. To address this issue, researchers should explore available institutional support, OA publishing funds, or waivers and discounts offered by institutions or by some publishers for scholars from low and middle-income countries. Authors can also leverage institutional or subject repositories to deposit post-peer-reviewed versions of their work [69], or use open preprint servers such as arXiv (https://arxiv.org), bioRxiv (https://www.biorxiv.org), EcoEvoRxiv (https://ecoevorxiv.org), medRxiv (https://www.medrxiv.org), MetaArXiv (https://osf.io/preprints/metaarxiv), or PsyArXiv (https://osf.io/preprints/psyarxiv), in conjunction with formal publication in a peer-reviewed journal. To navigate the self-archiving policies and restrictions of different journals, researchers can consult the Sherpa/RoMEO database (https://v2.sherpa.ac.uk/romeo/), which provides a comprehensive listing of publisher policies regarding the sharing of pre-prints, post-prints, and other versions of published articles.

Of note, open access is not just about removing financial barriers, it is also about enabling the reuse and distribution of content. Thus, researchers should familiarize themselves with the different types of Creative Commons licenses and, to the extent that it is possible, choose one that aligns with how they want their work to be used [70]. Researchers should also consider providing plain language abstracts or summaries of their meta-analyses. These serve as crucial tools for making complex research findings accessible to audiences across disciplines and to the general public. These summaries should be written in clear, jargon-free language, avoiding technical terms or disciplinary-specific terminology that may hinder comprehension. The focus should be on distilling the key findings, implications, and practical relevance of the meta-analysis in a concise and easy-to-understand manner. Together, these steps can help advance the reach and impact of researchers’ findings within the scientific community and the public at large.

## Tip #9: Promote your findings

Often considered secondary or even trivialized, promotion is a critical step to ensure that the synthesized evidence reaches a diverse audience, including other researchers, practitioners, policymakers, and the public [71]. Historically, promoting one’s findings has often taken the form of presentations at conferences, workshops, and webinars to reach academic and professional communities directly.

In the digital age, there are multiple additional avenues for promoting research findings. Social media platforms offer vast networks for sharing results rapidly and engaging with a global community [72], whereas academic networking sites provide forums for researchers to connect and share full-text publications with peers [73]. Blogging and podcasting are effective mediums for explaining the significance of meta-analysis findings in a more accessible language, thus bridging the gap between complex research and public understanding [74]. Infographics and short videos can also be used to convey key messages visually, making the information more digestible and shareable [75].

Engaging with traditional media by issuing press releases or coordinating with university media teams can also amplify the reach of research findings to a broader audience and may lead to coverage by journalists and influencers [76]. Researchers should emphasize the open and transparent nature of their work, highlighting the availability of data, materials, and analysis scripts for public scrutiny and reuse. In this context, plain language summaries as discussed in the previous tip can help broader promotion and dissemination efforts, and amplify the reach and impact of research findings. Together, these steps ensure researchers not only enhance the visibility and application of their work, but also fulfill their responsibility to contribute to evidence-informed decision-making in society.

### Integration with existing guidelines

To facilitate implementation of these 9 tips, we have developed an open meta-analysis checklist (https://osf.io/k8aqx/). The proposed checklist is intended to integrate seamlessly with prevailing reporting guidelines and best practices in the field. It complements and extends the widely adopted PRISMA 2020 statement for transparent reporting of systematic reviews and meta-analyses [6]. While PRISMA focuses on essential reporting elements, our checklist provides supplementary guidance on open science practices spanning protocol development, reproducibility, dissemination, and post-publication promotion.

Furthermore, the checklist aligns with the PRIOR statement [77] on making all parts of the research cycle publicly accessible. Its emphasis on preregistration, open data/code, and open-access publishing map directly to the core tenets outlined by the PRIOR statement.

Importantly, the checklist also upholds the FAIR principles [13] by advocating for practices that enhance the findability, accessibility, interoperability, and reusability of meta-analytic outputs. Recommendations such as use of version control, posting analysis scripts, and clear data documentation all serve to maximize the FAIRness of meta-analytic research products.

In collectively promoting open and transparent workflows, responsible data stewardship, and the development of accessible knowledge resources, the open meta-analysis checklist provides an actionable complement to these foundational guidelines and principles. It offers a tailored operationalization for embracing open science in the domain of meta-analysis.

## Conclusions

With these 9 quick tips, researchers can ensure their meta-analyses adhere to open science principles and best practices, promoting transparency, reproducibility, and accessibility. The tips also increase the likelihood a meta-analysis will stand the test of critical evaluation [78,79] and contribute meaningfully to the collective body of knowledge—though it is important to recognize that open practices alone do not guarantee a high-quality or impactful meta-analysis. The value and contribution of a meta-analysis to the collective body of knowledge also depend on the rigor of the methodology, the quality of the included studies, and the relevance of the research question being addressed. Beyond academic rigor, embracing these tips is a commitment to an open science ethos that values the dissemination and democratization of information: As meta-analysis continues to shape our understanding across various fields, adherence to these principles will facilitate a more collaborative, accessible, and innovative research environment, where knowledge can flourish unfettered by traditional barriers, and findings can be used to their fullest potential by all members of society.

There remain, however, areas that require further development and research. One key need is the creation of more user-friendly, integrated tools that seamlessly combine various open practices, from protocol development and preregistration to data extraction, analysis, and reporting, within a unified ecosystem. Such tools could lower barriers to entry and facilitate wider adoption of open meta-analytic workflows. Relatedly, there is a need for more comprehensive training resources and educational initiatives to equip researchers with the skills required for conducting open, reproducible meta-analyses [59].

Furthermore, as AI and machine learning capabilities advance, their responsible integration into meta-analytic processes must be carefully explored. New AI-based methods are emerging that could revolutionize and streamline various stages of the meta-analytic process. For example, tools like Abstrackr [80] use natural language processing to assist in the initial screening of literature search results, potentially accelerating study selection. AI-based text mining and data extraction approaches, such as those implemented in tools like RobotReviewer (https://robotreviewer.net), could help automate parts of the data extraction process from included studies. As these methods continue to evolve and become more accessible, developing best practices and guidelines for leveraging them while maintaining human oversight and methodological rigor will be crucial for harnessing their potential efficiency gains without compromising scientific integrity.

More generally, continued research is needed to evaluate the real-world impacts of open meta-analyses on scientific progress, evidence-based decision-making, and public trust in research. Empirical investigations into the adoption rates, challenges, and tangible benefits of these practices can inform further refinements and drive wider acceptance within the research community and beyond.
